# Percutaneous transhepatic biliary drainage (PTBD) in patients with dilated vs. nondilated bile ducts: technical considerations and complications

**DOI:** 10.1007/s00330-020-07368-6

**Published:** 2020-10-13

**Authors:** Federico Pedersoli, Anja Schröder, Markus Zimmermann, Maximilian Schulze-Hagen, Sebastian Keil, Tom Florian Ulmer, Ulf Peter Neumann, Christiane K. Kuhl, Philipp Bruners, Peter Isfort

**Affiliations:** 1 Department of Diagnostic and Interventional Radiology, University Hospital RWTH Aachen, Pauwelsstraße 30, 52074 Aachen, Germany; 2 Department of General, Visceral and Transplant Surgery, University Hospital RWTH Aachen, Aachen, Germany

**Keywords:** Cholestasis, Bile ducts, Cholangiography, Biliary tract neoplasms, Percutaneous transhepatic biliary drainage

## Abstract

**Objectives:**

The aim of this study was to compare success, technical complexity, and complication rates of percutaneous transhepatic biliary drainage (PTBD) in patients with dilated vs. nondilated bile ducts.

**Methods:**

In a retrospective analysis, we evaluated all consecutive PTBD performed in our department over a period of 5 years. Technical success, technical data (side, fluoroscopy time, radiation dose, amount of contrast media, use of disposable equipment), procedure-related complications and peri-interventional mortality were compared for patients with dilated vs. non-dilated bile ducts. Independent *t* test and χ^2^ test were used to evaluate the statistical significance.

**Results:**

A total of 253 procedures were performed on 187 patients, of whom 101/253 had dilated bile ducts and 152/253 not. In total, 243/253 procedures were successful. PTBD was significantly more often successful in patients with dilated vs. nondilated bile ducts (150/153 vs. 93/101; *p* 0.02). Overall complication rate (13%) did not differ significantly between patients with dilated vs. nondilated bile ducts. Procedures in patients with normal, nondilated bile ducts were associated with a significantly higher rate of post-interventional bleeding (5/101 vs. 0/152). Mean fluoroscopy time (42:36 ± 35:39 h vs. 30:28 ± 25:10 h; *p* 0.002) and amount of contrast media (66 ± 40 ml vs. 52 ± 24 ml; *p* 0.07) or use of disposables were significantly higher in patients with nondilated ducts. A significantly lower fluoroscopy time and amount of contrast medium were used in left hepatic PTBD.

**Conclusion:**

Despite the higher technical complexity, PTBD with nondilated bile ducts was associated with similar overall complication rates but higher bleeding complications compared with PTBD with dilated bile ducts.

**Key Points:**

*• PTBD was associated with similar overall complication rates in patients with dilated vs. nondilated bile ducts.*

*• Although overall complication rates were low, PTBD in patients with nondilated bile ducts was associated with a higher incidence of post-interventional bleeding.*

*• PTBD in patients with nondilated bile ducts is technically more complex.*

## Introduction

Percutaneous transhepatic biliary drainage (PTBD) is the gold standard treatment for patients in whom endoscopic retrograde cholangiography is unsuccessful or not possible [1–3].

The main indication for PTBD is often caused by tumors or (post-) inflammatory strictures, leading to cholestasis [4]. Moreover, an increasing number of PTBD are performed in patients with nondilated bile ducts, mainly to treat post-surgery bile leak in the site of the biliodigestive anastomosis [2, 5, 6].

The most severe complications of PTBD are pancreatitis, hemorrhage, fistulae between the bile duct and hepatic artery or portal vein, pseudoaneurysms, bile leaks, and transpleural punctures with risk of pneumothorax or hematothorax [7–9] and occur in 8.6–22% [10, 11] of the procedures. PTBD in patients with nondilated bile ducts are described to have a higher complication rate and a lower success rate compared with patients with dilated bile ducts [5, 12]. Nevertheless, these data are still under debate, with more recent studies suggesting a comparable technical success and complication rate [11].

The aim of the present study is to compare the efficacy and safety of PTBD in patients with dilated and nondilated bile ducts.

## Materials and methods

### Study population

Approval for this study was waived by the institutional review board (EK 385/19).

We conducted a retrospective analysis of patients who underwent PTBD procedure in our department during the last 5 years (January 2014–December 2019). Patients were dichotomized in a dilated vs. nondilated group based on pre-procedural CT and intraprocedural sonography. Dilated bile ducts were considered to be present when the diameter of an intrahepatic peripheral bile duct exceeded 2 mm, or in case the diameter of the bile duct was larger compared with the accompanying portal vessel.

Patient data (age, gender), indication for treatment, side of the drainage (left/right), and peri-interventional laboratory parameters (full blood count, CRP, γ-GT, bilirubin, GOT, ALT, AST) were collected. Fluoroscopy time, radiation dose, contrast media used and number of needles used were collected and considered an indirect measurement of the length and complexity of the procedure. Technical success was defined as the placement of PTBD with the distal end in the small intestine by means of one procedure. Complications were classified according to the CIRSE classification system [13]. Complications were considered to be clinically significant when they were graded as ≥ 3 on this scale.

The presence of cholangitis was determined either through clinical findings (e.g., fever) or, in asymptomatic patients, through microbiological assessment of the bile and/or through a rise of laboratory parameters indicative of infectious disease by at least 20% on days 0, 1, and 3 after the procedure

### Technique of PTBD

**Fig. 1:**
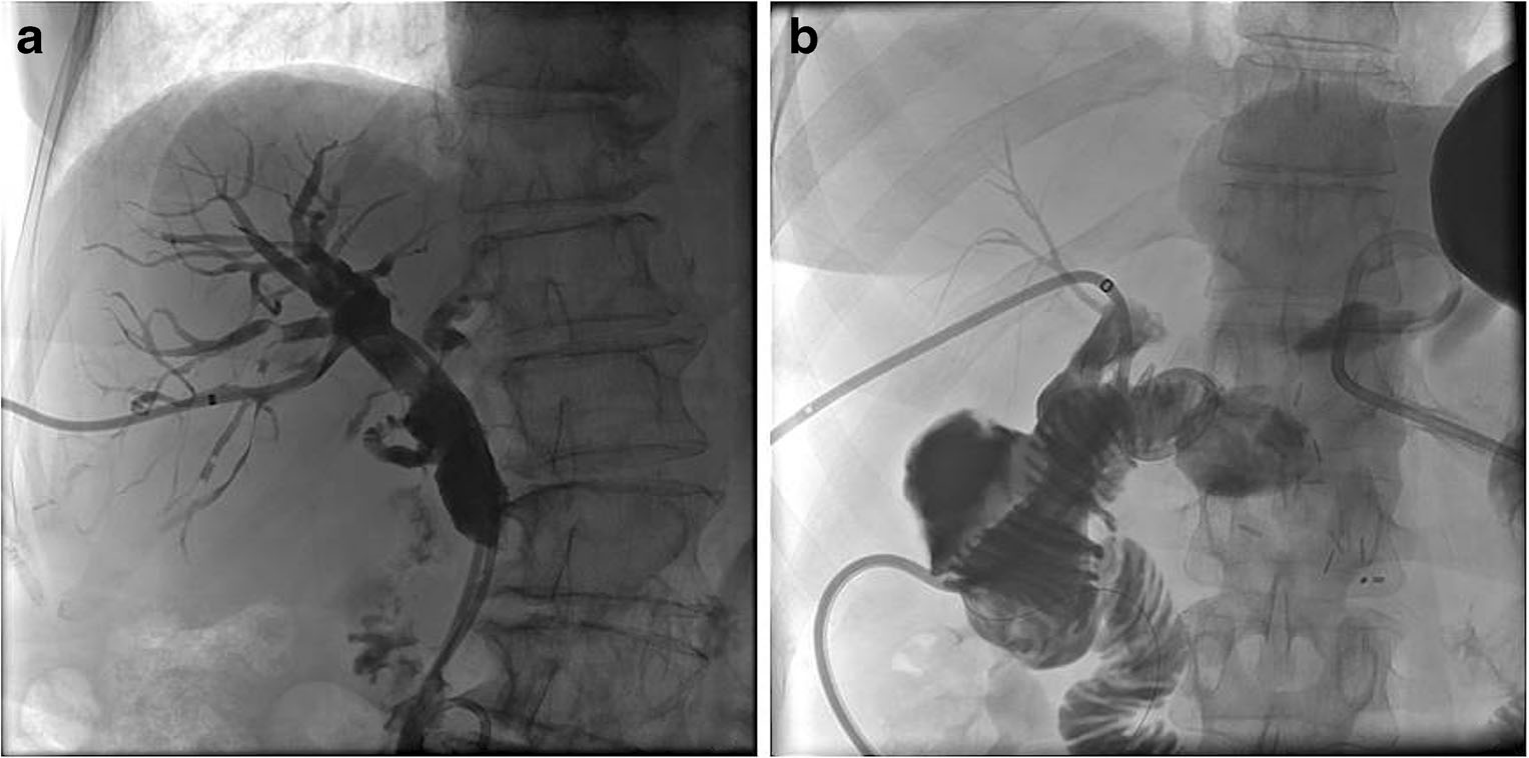
**a** PTBD to treat bile duct stenosis due to pancreatic cancer. **b** PTBD in a patient with insufficiency of the biliodigestive anastomosis after pancreatoduodenectomy for cholangiocarcinoma

The procedures were performed in an angio-suite (Artis zeego eco or Artis zee, Siemens Healthcare) after local anesthesia (10 ml Mepivacaine 1%, Meaverin, PUREN Pharma GmbH & Co. KG) as well as analgesia (7.5–15 mg piritramide, Dipidolor®, Piramal Critical Care Deutschland GmbH). Before the intervention, liver sonography was performed to plan the procedure. If possible, a right-sided approach was preferred. Only in cases of left-sided intrahepatic biliary obstructions, a left-sided approach was used. A peripheral branch of the bile ducts was punctured with a 22G Chiba needle (Cook Medical) under sonography and fluoroscopy guidance. In case of nondilated bile ducts, the punctures were performed close to the peripheral branches of the portal vein. The core needle was then removed, and a mixture of NaCl solution and iodine contrast (Ultravist 300, Bayer AG) was carefully injected while withdrawing the needle until bile ducts were visible. In case of difficult puncture, if a central duct was punctured first, the needle was left in place and contrast medium injected to opacify the peripheral ducts, which were then punctured using a second needle. If a percutaneous drainage was already in place, i.e., in case a biliary drainage had been inserted during surgery or for biloma drainage and in case that the drainage communicated with the intrahepatic biliary system, it was up to the interventionalists’ discretion to use it to opacify the biliary system, in order to facilitate the puncture. In case of successful puncture, a 0.018″ nitinol guidewire (Cook Medical) was inserted and thereafter a 4F introducer sheath (Neff Percutaneous Access Set, Cook Medical) was introduced in the Seldinger technique. Thereafter, a 0.035″ guidewire (Terumo Corporation) was advanced in the small bowel. If necessary, a 4F hydrophilic catheter (different shapes; Glidecath, Terumo) was used to guide the wire. Thereafter, the wire was exchanged to a stiff 0.035″ guidewire (Amplatz, Boston Scientific Corporation) and a 8.5F internal external drainage (Cook Medical) was placed (Fig. 1).

### Statistical analysis

Quantitative measurements are expressed in means. Independent *t* test was used to compare the quantitative parameters in dilated vs. nondilated group as well as to compare PTBD of the left vs. the right liver. Datasets collected in nominal scale were evaluated by the χ^2^ test. Statistical significance was considered with a *p* value ≤ 0.05.

## Results

### Patient data

A total of 253 interventions in 187 patients were included in the study, 101/253 interventions (40%) in nondilated bile ducts and 152 interventions (60%) in dilated bile ducts. In total, 113/187 (60%) were men, and 74/187 (40%) women. Mean age was 67 years. In 47 of 101 procedures with nondilated bile ducts, a drainage was in place that could be used to guide to puncture by means of bile duct opacification.

### Indications

Indications of PTBD were bile duct stenoses (151/253) or bile leakage (102/253). A total of 127/151 patients who received PTBD to treat a stenosis presented with dilated bile ducts, whereas 24/151 had no dilated bile ducts. Causes for bile duct stenoses were represented by obstructive tumors (95/151) or strictures secondary to biliary tract surgery (42/151). Fourteen out of 151 patients presented stenoses for other benign reasons. Seventy-seven out of 102 patients who received PTBD to treat a leakage had nondilated bile ducts whereas 25/102 had dilated ducts. Bile leakage occurred in patients with insufficiency of biliodigestive anastomosis (64/102) or as complication of a different biliary surgery, e.g., cholecystectomy (13/102).

A total of 152/253 of all PTBD implants were post-surgical. A total of 112/253 of interventions occurred in patients who suffered from cholangiocarcinoma. Details regarding indications for PTBD are reported in Table 1.

**Table 1:** Indications for PTBD

Indication	Nondilated bile ducts (*n* = 101)	Dilated bile ducts (*n* = 152)	*p* values
Stenosis	24	127	
Stenosis of bile ducts after biliary surgery	7	35	0.001
Stenosis by tumor compression	14	81	0.001
Stenosis by benign compression	1	5	0.239
Liver graft failure/primary sclerosing cholangitis	2	6	0.381
Leak	77	25	
Bile leak after biliodigestive anastomosis	52	12	0.001
Bile leak after other biliary surgery	5	8	0.912
Biliary abscess/biloma	20	5	0.001

### Efficiency of intervention

The procedure was technically successful in 242/253 cases (96%) and unsuccessful in the remaining 11 cases (4%). Unsuccessful interventions occurred significantly more frequently in patients with nondilated (8/101, 8%) vs. dilated bile ducts (3/152, 2%) (*p* = 0.02). In 5/11 unsuccessful cases, the correct placement of the drainage succeeded in the second intervention; one further case (1/11) required a third attempt for successful drainage. In 3/11 cases, a stent was placed during a second ERCP, which was performed after an unsuccessful PTBD, despite a first ERCP was primarily unsuccessful and PTBD had been performed as bailout treatment. In 2/11 cases, the intervention had to be stopped due to lack of cooperation or due to hypoglycemia. In both cases, the intervention could be carried out on the following day after an appropriate pre-treatment of the patient.

### Complications

Complications occurred in 34/253 (13 %) interventions, with 29/34 clinically significant complications. A complication rate of 14/101 (13.9 %) was observed in interventions with nondilated bile ducts and 15/152 (9.9 %) for interventions in dilated bile ducts, showing no statistically significant difference (*p* = 0.33). Post-interventional cholangitis was more frequent in patients with dilated bile ducts compared with that in non-dilated bile ducts (9/152 vs. 2/101; *p* = 0.132). All cases of intervention-associated cholangitis could be treated with antibiotic therapy.

**Fig. 2:**
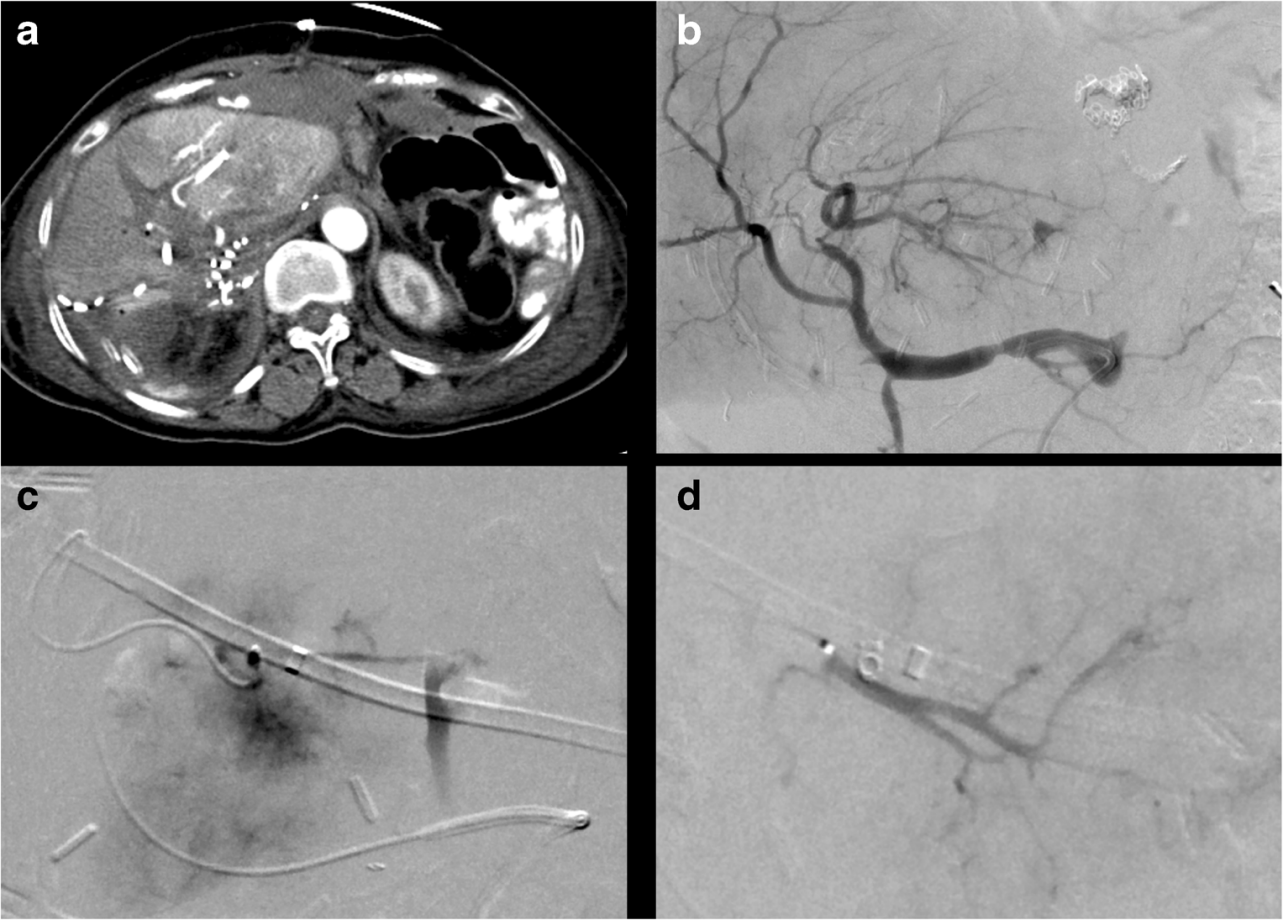
Case of a 66-year-old patient who underwent right hemihepatectomy to treat an intrahepatic cholangiocarcinoma. On day 12 after surgery, the patient developed an insufficiency of the biliodigestive anastomosis, which was treated by PTBD. Two days after the procedure, the patient presented clinical signs of hypovolemia. **a** CT acquired during the arterial contrast phase exhibited an arterial bleeding with peri-hepatic hematoma along the site of insertion of the PTBD in segment II. **b**, **c** DSA revealing the site of bleeding. **d** DSA after successful transarterial coil embolization of the bleeding

Hemorrhage occurred exclusively after interventions in nondilated bile ducts (5/101) requiring a blood transfusion in 2/5 cases. In further 2/5 patients, coil embolization of pseudoaneurysms of the hepatic artery was necessary to manage the hemorrhage (Fig. 2). One patient died because of hemorrhagic shock. This patient received a biliodigestive anastomosis to repair an iatrogenic bile leakage, which caused a septic shock. Thirteen days after the intervention, the patient underwent coil embolization of a pseudoaneurysm of the right hepatic artery because of active bleeding. Immediately after the intervention bleeding signs persisted and a laparoscopy revealed a diffuse venous peri-capsular bleeding which was treated with peri-hepatic packing. The patient died 2 days thereafter because of multiorgan insufficiency. Complications are shown in Table 2.

**Table 2:** Type and rate of complications observed in patients with nondilated vs. dilated bile ducts

Procedure-related complication	Nondilated bile ducts	Dilated bile ducts	*p* values
Overall complications	17 (16.9%)	17 (11.2%)	0.197
Relevant complications	14 (13.9%)	15 (9.9%)	0.329
Cholangitis	2 (1.9%)	9 (5.9%)	0.132
Bleeding	5 (4.9%)	0 (0%)	0.006
Pneumothorax	1 (1%)	1 (0.7%)	0.770
Pseudoaneurysm	2 (2%)	2 (0.8%)	0.678
Fistula	2 (2%)	2 (0.8%)	0.678
Biloma/biliary leak	3 (3%)	2 (0.8%)	0.355
Other	2 (2%)	1 (0.7%)	0.341

### Technical parameters of intervention

The mean fluoroscopy time was 00:42:36 ± 00:35:39 h for nondilated bile ducts and 00:30:28 ± 00:25:10 h for dilated bile ducts (*p* 0.002). Mean radiation dose was 18651 ± 17689 cGy cm^2^ for nondilated bile ducts and 14670 ± 16099 cGy cm^2^ for dilated bile ducts (*p* 0.07). For nondilated bile ducts, an average of 66 ± 40 ml contrast medium was used, and for dilated bile ducts 52 ± 24 ml (*p* 0.001). A median of two needles (range 1; 6) were used in interventions in nondilated bile ducts compared with one needle (range 1; 5) in interventions in dilated bile ducts (*p* 0.002). In procedures with subsequent complications, a 33% higher fluoroscopy time (*p* 0.06), a 28% higher radiation dose, a 10% higher use of contrast media, and a greater number of needles (mean 2.21 vs. 1.79) (*p* = 0.062) were observed. A significantly lower fluoroscopy time (*p* 0.003) and a significantly lower amount of contrast medium (*p* 0.002) were used in patients with left hepatic PTBD. Mean radiation dose and the number of needles used were also lower in cases of left hepatic approach (110/253) but did not differ significantly from the right-sided approach. Also, this association did not differ between interventions in dilated vs. nondilated bile ducts.

## Discussion

In this study on 253 PTCD procedures, PTBD was confirmed to be an efficient and safe method for external-internal biliary drainage, with a technical success rate of 96%, which is in agreement with the expected success rates defined by the CIRSE guidelines [14].

We did observe a small but significant difference in technical success rate between PTCD in patients with dilated (98%) vs. nondilated (92%) bile ducts. Moreover, the mean fluoroscopy time, amount of contrast media, and the number of used needles were significantly higher in procedures on patients with nondilated bile ducts, reflecting the increased procedural difficulty in this cohort.

Kühn et al in 2008 and Weber et al in 2009 reported their experiences on PTBD in patients with dilated vs. nondilated bile ducts. They did not find a statistically significant difference between the two groups in terms of technical success rates [11, 12]. However, their overall success rate was lower than the one observed in our cohort, with 90% and 81% in patients with dilated and non-dilated bile ducts, respectively, in the report by Kühn [11], whereas these rates were slightly higher, i.e., 98% and 92%, in our cohort. Weber et al did not distinguish between the success rates in dilated vs. non-dilated bile ducts; moreover, Weber defined technical success as the correct placement of a drainage tube anytime, i.e., possibly after multiple attempts [12].

We did not find a statistically significant difference of the rate of clinically relevant complication in patients with dilated (9.9%) vs. nondilated bile ducts (13.9%). This is in good agreement with the results published by Kühn et al, who, in a cohort of 71 patients undergoing 97 PTCD procedures, observed a complication rate of 7.3% vs. 10.0% [11]. By way of contrast, in the study published by Weber et al on 419 patients, a statistically significantly higher complication rate was observed in 6.9% vs. 14.5% of patients with dilated vs. nondilated bile ducts [12]. A possible explanation for this discrepancy could be the different technical implementation. In our study, as well as in the study of Kühn et al and in other studies [2, 11, 15], supplementary techniques to opacify the bile ducts in patients with nondilated bile ducts were used, such as retrograde opacification via pre-existing biloma drainages, or through the additional puncture of a central duct. In our cohort, a substantial number of procedures (102/253; 40%) were performed on patients with bile leakage, whereas this type of patients was not included in Weber’s cohort and represented only a minority (17 patients) in Kühn’s report. We believe that this can contribute to the observed discrepancy with Weber’s results.

We also compared fluoroscopy time needed to complete the procedure and found that significantly more time was needed in patients with nondilated bile ducts; accordingly, also radiation dose was higher, although this did not reach statistical significance.

Bleeding complications were observed in 2% of all patients and were observed only in patients with nondilated bile ducts. The higher number of transhepatic needle passes required to successfully puncture a non-dilated bile duct is likely the most important reason for this potentially life-threatening complication [5, 10, 11]. A close post-interventional follow-up and immediate treatment of possible post-procedural bleeding is advocated to reduce risks associated with this complication.

Cholangitis was observed in 4% of our patients, 82% of whom had dilated bile ducts, suggesting the presence of cholestasis as a predisposing factor. Still, this rate compares favorably with the published incidence of post-interventional cholangitis which has been shown to vary widely between 1.67 and 35% [2, 12, 16]. In our study, no infectious complications like sepsis or pancreatitis were observed. The transpapillary position of the prosthesis by the internal/external drainage has been associated with a higher incidence of cholangitis [17, 18], likely because it facilitates migration of bacteria from the foregut into the biliary system. This possible side effect needs to be balanced against the advantage of restoring the physiological flow of bile to the gut. In addition, from a technical perspective, with internal/external drainages, the distal tip of the drainage is anchored in the foregut to improve stability and reduce the risk of dislocation.

In good agreement with current literature [19], we found that a left hepatic approach was associated with a significantly shorter fluoroscopy time and reduced need of contrast medium. This aspect is interesting especially if the access path is not determined by the site of occlusion or the individual patient anatomy. Even if associated with a higher radiation exposure to the hand of the operator [20], a left hepatic approach may be favored, especially in the already technically difficult setting of patients with nondilated bile ducts.

Mean radiation dose associated with PTCD procedures in patients with dilated (14670 cGy cm^2^) and nondilated (18651 cGy cm^2^) bile ducts was substantially above the national diagnostic reference levels published in 2019 [21], which advise a radiation dose below 4300 cGy cm^2^ for initial PTBD placement. The mean doses observed in our patients were, however, lower compared with published radiation dose associated with PTCD placement [22, 23]. Moreover, our data were based on 253 interventions, which are comparable to the 256 interventions considered in the study which leaded to the formulation of the diagnostic reference levels.

However, in our study, we only included patients who underwent percutaneous insertion of a biliary drainage, whereas in the manuscript regarding the national diagnostic reference [21], primary PTBD referred to patients who received an endoprosthesis (210 cases) or metal stent (30 cases), clearly a different setting compared with ours. Moreover, in our cohort, 40% of patients were in a post-surgical situation; in the majority of these cases, it is essential to exclude presence of biliary leakage—which, in turn, requires the acquisition of multiple runs of digital subtraction angiograms, leading to higher radiation dosages. The need to acquire confirmatory angiograms from multiple projections will also be higher in post-surgical patients who typically harbor surgical clips, other drainages, etc. that may obscure the region of interest.

Limitations of this study are its retrospective design. Our analysis included patients who underwent PTBD for a wide spectrum of diseases. Moreover, post-surgical patients who required PTBD were often in critical clinical situation, a fact that will confound the results of follow-up.

In conclusion, even if more complex from a technical point of view, PTBD in patients with nondilated bile ducts was associated with a similar incidence of overall complications. Still, PTBD in patients with nondilated bile ducts was associated with a small, but significantly higher risk of bleeding complications compared with PTBD in patients with dilated bile ducts. This should be taken into account in the peri-interventional management of this specific subgroup of patients.

## Abbreviations

PTBD: Percutaneous transhepatic biliary drainage
